# From genetic counseling to “genomic counseling”

**DOI:** 10.1002/mgg3.45

**Published:** 2013-11-11

**Authors:** Kelly E Ormond

**Affiliations:** Department of Genetics and Stanford Center for Biomedical Ethics, Stanford University School of MedicineStanford, CA, 94305-5208

Genetic counseling is “the process of helping people understand and adapt to the medical, psychological, and familial implications of genetic contributions to disease.” Traditionally, this process includes collecting and interpreting the family and medical history, risk assessment, a comprehensive educational process for potential genetic testing, informed consent, and psychosocial assessment and support (National Society of Genetic Counselors' Definition Task Force et al. 2006). While genetic counseling falls within the scope of many health care professionals, clinical geneticists (physicians) and masters level genetic counselors have been working in the United States for more than 40 years, providing genetic counseling primarily for single-gene conditions. Debate about what “genomic counseling” will include and who will practice it has been fueled by the transition from single-gene focused genetic counseling and testing to a full genomic medicine approach. The routine incorporation of genomic medicine will likely induce differences in the *scope*, *approach* and *process* of genetic counseling (Table 1). In this commentary, I will discuss the several areas where practice will likely change as we move toward “genomic” counseling, with a focus on the unique skills and roles that genetic counselors and clinical geneticists provide.

**Table 1:** Changes that will impact the transition to “genomic counseling.”

Scope	Approach	Process
Increased number of conditions included in testing	A move from testing based on a specific clinical indication to broader testing approaches	Importance of bioinformatics and EMR to facilitate clinical incorporation of genomic results effectively
Increased number of “positive” and uncertain results, and overall increased number of disclosed results	Balancing increased uncertainty around variable (and changing) clinical validity and utility of genomic results	“Who, what and when” aspects of genomic testing and counseling still under debate
Increased time spent with clinicians	A move from a diagnosis focused approach toward a preventative approach where genomics influences both medical and personal aspects of healthcare	A likely but controversial shift from the historical focus on patient autonomy and nondirectiveness toward a more preventative health approach emphasizing behavior change

EMR, electronic medical record.

## The Family History and Risk Assessment

A cornerstone of a genetic assessment is obtaining and interpreting the family history, whether by phone, through a paper or web-based interface, or as part of a clinic visit. Traditionally, the personal and family medical histories have been used to develop a differential diagnosis, to identify and quantify risk for family members, and to select the appropriate test and proband (Pyeritz 2012). Taking an oral family history orally has also been useful in learning about the health beliefs and risk perceptions of family members and assessing communication patterns related to disclosure of genetic information (Bennet 2004). This is especially important when there is a family history of inherited disease, and individuals have experiential knowledge, and often come in with knowledge of the lived experience, and often strong feelings about the condition, their own potential risks and whether they wish to obtain predictive knowledge about genetic risk. As discussed later, this leads to self-selection in those who ultimately undergo predictive testing and when they choose to be tested.

Further emphasizing the importance of the family history, whole exome sequencing (WES) and whole genome sequencing (WGS) may not provide full coverage of critical genes, and the family history allows the genetic team to generate a differential diagnosis and order more sensitive genetic testing if necessary. As WES/WGS increases in sensitivity, however, family history will be critical for prioritizing variant analysis and adding perspective (pretest probability) to the interpretation of susceptibility genes and findings “incidental” to the clinical indication for testing. (e.g., Ashley et al. 2010; Dewey et al. 2011). Additionally, family history will provide a roadmap for evaluating how variants of unknown significance segregate with affected family members. Genetic counselors and clinical geneticists are well positioned for obtaining tailored family histories, using it to provide anticipatory guidance regarding what a genomic study may identify, identifying the relevant individual and family health beliefs, and supporting family communication about genetic risks, especially until a time when genome sequencing becomes the ubiquitous part of medical care.

## Education and Informed Consent

The traditional approach to genetic counseling for single-gene disorders is highly education focused, and genetic counseling sessions can last 30–90 min or more. A recent practice analysis suggests typical genetic counseling sessions can include (but are not limited to): a review of general genetic principles, modes of inheritance, family/individual specific risk assessment, an in depth discussion of the diagnosis and natural history, potential testing options, and case management for the condition occurring within the family or for which they are at risk (Hampel et al. 2009). Ideally, in a genetic counseling session, a psychoeducational and person-centered approach allows the information to be tailored to the person's understanding level, culture, and personal context. As genomic medicine progresses, genetic counselors and geneticists remain well positioned as experts in the benefits and limitations of the technology and the clinical implications of Mendelian and non-Mendelian genetic conditions. Additionally, genetic counselors have expertise in risk communication, genetic and health literacy, and numeracy. It remains to be seen if the genomic revolution will require genetics practitioners to subspecialize in order to master the increasing amount of genetic information, or to become clinical generalists in order to address the full range of information a genome will provide. I suspect we will need both to navigate the future genomic revolution.

Given the issues in “scope” mentioned in Table 1, pretest informed consent for genomic testing can no longer maintain the traditional “comprehensive” educational approach for single gene disorders described above, as patients neither have the ability nor desire to comprehend that volume of information. We are seeing this already in clinical practice; as “panel tests” become more commonly used for specific clinical indications, many genetic counselors have already transitioned their pretest informed consent discussions to broadly explain the indications for testing, the focus of the test, the range of findings that may result, and the potential benefits and limitations of testing. However, result sessions remain focused on the disorder and its potential management when a pathogenic variant is identified, emphasizing testing limitations, additional testing options, and residual risks when a variant of uncertain significance (VUS) or no variant is identified. Educationally, this benefits the patients, who may be overwhelmed by the sheer volume of pretest information and find many of the clinical conditions personally irrelevant until a result is demonstrated in their family. As genetic counselors develop variations on the concept of “generic consent” (Elias and Annas 1994), research should be performed to examine not only what patients hypothetically believe they want to know in order to consent to genome testing, but also retrospectively, to examine what approaches are most effective and useful for patients in deciding whether to undergo genetic testing, and which variables most influence the desired pretest information.

A new challenge in genetic counseling will be discussing which incidental findings, if any, will be assessed and returned to patients, creating a plan for such return of results, and documenting the patients' decline of such information if applicable. As part of these discussions, it will be important to remember that patients may have low familiarity and few formed opinions about the “lived experience” for this wide range of conditions, which could make it more challenging to make informed decisions in this area.

Finally, genetic counseling has developed models of service delivery that go beyond the traditional “face to face” approach – these include phone or telemedicine counseling, and both static and interactive e-learning approaches, sometimes to augment “live” genetic counseling and sometimes as a stand-alone education approach,. In recent years, direct-to-consumer (DTC) approaches have evolved from these educational approaches. Genetic counselors and geneticists are trained in patient education and will continue to find roles in developing interactive educational content across many of these venues. I encourage research on the effectiveness of these approaches, and clinicians may need to have multiple educational approaches available to address the varied learning styles of patients.

## Ordering and Interpreting Genetic Tests

Genetic counselors and clinical geneticists have traditionally served as the “genetic experts,” in medicine, often in a consulting role despite being a primary medical specialty. In the past decade, genetic counselor roles have expanded significantly from the original prenatal and pediatric genetic counselor roles. A significant minority have taken on “laboratory genetic counselor” roles (National Society of Genetic Counselors 2012), serving a critical role in assuring that the proper genetic testing is ordered on the correct person, and that the ordering physicians understand the result and its implications (Scacheri et al. 2008). Clinical genetic counselors in specialty areas (e.g., oncology, cardiology, neurology) often serve as the primary experts with regards to genetic testing while working in conjunction with the nongeneticist specialist physician. These role expansions are likely to continue as genomic medicine matures. Across all specialties, but particularly in cancer and cardiology genetics where VUS results are frequent outcomes to panel-based genetic testing, genetic counselors have had to understand variant interpretation and, in many cases, perform manual annotation of variants reported by a laboratory. Genetic counselors and geneticists already sit on interpretation panels for determining what warrants disclosure, and will remain experts in this area. This role will become increasingly relevant for all genetic counselors, whether they work directly with patients or not, and our training and continuing education processes will need to ensure that all genetic counselors are proficient in variant interpretation and understand the laboratory and bioinformatics processes.

## Psychosocial Support and Adjustment

One thing that separates genetic counselors from other health professionals with expertise in genetics is their stated focus on the psychosocial adaption to genetic conditions or genetic risk (Biesecker and Peters 2001). It will remain critical that genetics counselors help patients personalize their choices about whether and when to undergo genomic testing, and the implications of learning genomic variation, along with all its concomitant medical and social implications as individuals and within their family structure. I will discuss below two areas where I believe the genetic counselor's approach to psychosocial counseling may change as we move toward genomic medicine, based both on the more generalized testing approach and the hopes for preventative genomic medicine.

We have moderately good data about the psychosocial impact of learning carrier status (Lewis et al. 2011) or predictive risk for a highly penetrant genetic condition (e.g., Evers-Kiebooms et al. 1997; Bleiker et al. 2013). Data are also emerging on testing children for adult onset conditions ranging from familial adenomatous polyposis (FAP) (e.g., Michie et al. 2001; Codori et al. 2003) to breast cancer (Bradbury et al. 2008) to carrier status for autosomal recessive diseases. However, the vast majority of this data come from a population of individuals who were aware of their family history and opted for predictive genetic testing on the basis of pretesting psychological features, social support, and expectations of how the results may impact them. Self-selection also varies by disease characteristics; testing uptake for certain highly penetrant cancers where surveillance is available hoovers near 50% of the at-risk population, whereas for Huntington disease it can be below 20%. (Evers-Kiebooms et al. 1997). Limited data regarding the receipt of low penetrance genotyping risk data suggest that for most individuals, neither anxiety nor depression is clinically increased in the short or long term (e.g., Bloss et al. 2011). But data regarding the psychosocial responses of individuals who receive unexpected but highly penetrant genetic risk information (e.g., BRCA test results unexpectedly) from genome testing are limited (Francke et al. 2013, F. A. Dewey, M. Grove, C. Pan, B. A. Goldstein, J. Bernstein, H. Chaib, R. Goldfeder, C. Caleshu, K. Kingham, K. E. Ormond, T. E. Klein, M. Whirl-Carillo, K. Sakamoto, M. T. Wheeler, A. Butte, J. Merker, J. Ford, L. Boxer, J. Ioannidis, A. C. Yeung, A. Altman, T. L. Assimes, M. Snyder, E. A. Ashley, T. Quertermous, pers. comm.), and is biased by the fact that healthy individuals undergoing DTC genotyping and/or WGS are early adopters who may have specific psychosocial characteristics limiting the generalizability of this data. While these cases are likely to be rare, and a rigorous pretest family history may identify some high-risk individuals, more research is needed on the short- and long-term psychosocial implications of receiving such information.

Given the psychosocial implications of learning that one carries a highly penetrant condition that may have limited medical actionability, and given the worldwide history of eugenics and stigma associated with genetic conditions, there has been a strong focus on individual autonomy and non-directiveness around genetic testing decisions and future medical management (Weil et al. 2006). For those with a family history of a single-gene condition, a values-based decision-making approach toward genetic testing will remain its relevance in years to come. However, in the past decade, specialist genetic counselors have started to change toward more “directive” health promotional counseling, particularly in highly penetrant but medically actionable conditions such as cancer and sudden death cardiac conditions (e.g., Albada et al. 2013). This may strike some as a radical departure from nondirective genetic counseling until one reframes the approach in terms of providing patient-centered counseling that identifies relevant values, beliefs and barriers toward health behavior change, and then supports such change while respecting and supporting the patient's values. As such, health education and promotion becomes an important part of the genetic counselor's job, and in fact meets the definition of genetic counseling that we started with: “helping people understand and adapt to the … genetic contributions to disease” (National Society of Genetic Counselors' Definition Task Force et al. 2006). Data from early genome wide association (GWA) studies suggest a limited behavior change after genetic risk prediction for common complex disease (e.g., Bloss et al. 2011), but these studies were primarily conducted in a DTC setting with limited health provider intervention. The “promise of genomic medicine” has always been preventative health care; if we can find patient-centered ways to galvanize preventative health behaviors, we can empower a generation of patients toward better health. Genetic counselors are already well positioned to play a pivotal role in this area, but to do so, will need to become more familiar with health promotion models, apply them in practice and perform longitudinal outcomes studies to determine their utility and effectiveness.

The profession of genetic counseling has undergone many transitions since its inception over 40 years ago; it has expanded from a primarily pediatric and obstetric focus at a time when genetic testing did not even exist, into multiple medical specialties that have access to rapidly changing genetic tests. The unique skills and roles of clinical geneticists and genetic counselors will become even more paramount, and genomic counseling will evolve in ways that preserve the central tenets of values-based decision making for patients while also promoting patient health outcomes.
